# Hesperidin Inhibits Lung Cancer *In Vitro* and *In Vivo* Through PinX1

**DOI:** 10.3389/fphar.2022.918665

**Published:** 2022-07-01

**Authors:** Yang Yao, Mingyue Lin, Zhujun Liu, Mengyang Liu, Shiheng Zhang, Yukun Zhang

**Affiliations:** ^1^ Chongqing Key Laboratory of Development and Utilization of Genuine Medicinal Materials in Three Gorges Reservoir Area, Chongqing, China; ^2^ Department of Basic Medicine, Chongqing Three Gorges Medical College, Chongqing, China; ^3^ Ministry of Public Infrastructure, Chongqing Three Gorges Medical College, Chongqing, China; ^4^ Department of Pharmacy, Anhui Medical University, Hefei, China; ^5^ State Key Laboratory of Natural and Biomimetic Drugs, Department of Pharmacology, School of Basic Medical Sciences, Peking University, Beijing, China

**Keywords:** hesperidin, lung cancer, PinX1, cell senescence, natural products

## Abstract

New drugs or active leads with high efficiency and low toxicity are needed in the treatment of lung cancer. Natural products are an important source of anti-tumor drugs. At present, there are many molecular-targeted anti-tumor drugs derived from natural products or their derivatives for tumor treatment or in clinical trials. Hesperidin is a flavanone isolated from the Rutaceae plant lime *Citrus aurantium* L. or *Citrus sinensis* Osbeck. It has been considered to inhibit cancer cell viability *in vitro*. However, the effect of hesperidin on lung cancer and its underlying mechanism remain unclear. In this study, we found that the pinX1 expression level is closely related to overall survival and plays an important role in regulating lung cancer cell proliferation, migration, invasion, and senescence. More importantly, hesperidin significantly increased pinX1 protein expression, and knockdown pinX1 by its specific siRNA blocked the protective effects of hesperidin. Moreover, we also assessed that hesperidin at 100 mg/kg is safe *in vivo*. These findings showed that hesperidin is a potential therapeutic candidate for preventing the progression of lung cancer.

## Graphical Abstract

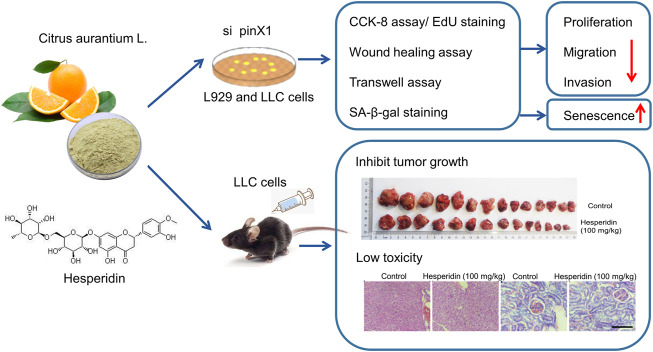

## Introduction

Lung cancer has been the most common cancer worldwide since 1985, both in terms of incidence and mortality. Globally, lung cancer is the largest contributor to new cancer diagnoses (1,350,000 new cases and 12.4% of total new cancer cases) and to death from cancer (1,180,000 deaths and 17.6% of total cancer deaths) (Ferlay et al., 2010; Dela et al., 2011). The current treatments for lung cancer mainly include chemotherapy, radiotherapy, and surgical treatment. However, chemotherapy and radiotherapy can cause various side effects in patients, and surgical treatment has certain risks. It is very necessary to find and develop new drugs or active leads with high efficiency and low toxicity benefits for lung cancer.

With the rise of the upsurge of returning to nature, more and more people are looking for active lead compounds from nature that can effectively prevent and treat human diseases. Therefore, searching for active natural products and actively researching the biological activity and molecular mechanism of anti-lung cancer cells are of great significance for preventing and treating lung cancer, as well as the development of innovative Chinese medicines. Hesperidin, a member of the flavanone group of flavonoids, can be isolated in large amounts from the rinds of some citrus species. Studies have shown that hesperidin has the effects of maintaining normal osmotic pressure of blood pressure, reducing blood vessel fragility, reducing human cholesterol content, lowering blood pressure, inhibiting cancer, anti-virus, and anti-allergic (Muhammad et al., 2019). Studies have also suggested that hesperidin can accelerate cancer cell apoptosis by promoting oxidative stress, and it can also protect against chemically induced liver cancer by inhibiting the classic PI3K/Akt pathway (Zhang et al., 2021). Previous studies also reported the exact protective effect of hesperidin in breast cancer (Hsu et al., 2021), cervical cancer (Pandey et al., 2021), lung cancer (Birsu et al., 2015), and prostate cancer cells (Ning et al., 2020). However, the anti-tumor effects of hesperidin *in vivo* and the underlying mechanism of hesperidin on lung cancer are still unclear.

Telomere is a cap structure at the end of eukaryotic chromosomes. The length of telomeres is closely related to cancer and cellular aging (Button et al., 2022). As cells age, telomeres shorten as the number of mitotic divisions increases. Telomerase activation has been well documented as one of the necessary factors for cell immortalization and transformation. It is reactive in most human cancer cells (Counter et al., 1992; Kim et al., 1994; Broccoli et al., 1995; Taylor et al., 1996). In tumors, increased telomerase activity caused tumor cells to escape from cell senescence. Therefore, inhibiting telomerase activity may be an anti-tumor mechanism. Telomerase activity is regulated by a variety of proteins (Tian et al., 2014). PinX1 has the unique property of directly interacting with the telomerase catalytic component TERT. PinX1 can also potently inhibit telomerase catalytic activity (Zhou et al., 2011). In the previous study, it has been found that pinX1 overexpression in human cancer cells shortens telomeres, induces crisis, and inhibits tumorigenicity, whereas pinX1 depletion plays the opposite role. Moreover, the ability of pinX1 to inhibit telomere elongation has been shown to be highly conserved in other organisms such as yeasts and rats (Liao et al., 2002; Banik et al., 2004; Lin et al., 2004; Oh et al., 2007). What is more, in our pre-experiment, we found that hesperidin inhibited cancer cell proliferation with an increase of pinX1 expression. However, nothing is known about whether hesperidin has any role in pinX1-mediated cell senescence.

This study will investigate the anti-lung tumor effect of hesperidin *in vitro* and *in vivo* as well as the role of pinX1 during this process. This study will discover new anti-lung cancer drug targets and active leads, which can provide information for the in-depth research and development of hesperidin and the development of innovative drugs, and it can also provide a solid theoretical basis for the development of the citrus peel industry.

## Materials and Methods

### Materials and Reagents

Hesperidin (>98%) was purchased from Aladdin Reagent Co., Ltd. (Shanghai, China). Unless otherwise stated, all reagents were purchased from Sigma (St. Louis, United States).

### Patients and Samples

A lung squamous cell carcinoma (SCC) tissue chip was purchased from Outdo Biotech (Shanghai, China), which contains 86 standardized lung SCC samples, with a median age of 60 years ranging from 42 to 77 years. All samples were from patients who underwent surgery for complete removal of cancer.

### Cell Culture

Lewis lung carcinoma (LLC) cells were purchased from ScienCell company (CA, United States) and cultured in 1640 medium (Gibco, CA, United States) with 10% (v/v) fetal bovine serum (FBS, Gibco), 20 mg/ml penicillin, and 20 mg/ml streptomycin and maintained at 37°C in a humidified atmosphere of 5% CO_2_.

### Cell Viability Assay

The cell counting kit-8 assay was performed using a cell viability assay kit (Dojindo Laboratories, Shanghai, China) to assess cell viability. Cells were seeded into 96-well plates at a density of 8,000 cells per well and allowed to grow for 24 h with or without different concentrations of hesperidin. At the specified time points, 10 μl CCK-8 was added to each well following incubation for 2 h, and cell viability was detected at a wavelength of 450 nm, using a spectrophotometer (Thermo Fisher Scientific, Inc., CA, United States).

### EdU Proliferation Assay

The EdU proliferation assay was performed as previously described (Han et al., 2022). Briefly, cells were seeded on a six-well plate and cultured under the indicated conditions overnight. The cell proliferation was measured using the incorporation of EdU with the BeyoClick™ EdU Cell Proliferation Kit with Alexa Fluor 488 according to the manufacturer’s instructions. Also finally, the cell nuclei were stained using DAPI.

### Wound Healing Assay

The cell migration ability was determined by seeding 1×10^6^ cells into six-well plates with the 1640 medium and 10% FBS. After 12 h, a wound was made to the confluent cell layer using a 200-μl pipette tip. Images of the wounded area were captured at 0, 24, and 48 h. Cell migration capacity was calculated by determining the wound opening area using ImageJ software.

### Cell Invasion Assay

Cell invasion assays were performed in triplicate in 24 transwell units with 8-µM filters coated with Matrigel (BD Biosciences, LA, United States). Each well was loaded with 1× 10^5^ cells. After incubation for 24 h, cells passing through the filters into the bottom wells were fixed in 100% methanol and stained with crystal violet. Images were taken under a microscope (IX51, Olympus), and cell numbers were counted using ImageJ software.

### β-Gal Staining

For detection of senescence *via* β-gal staining, cells were washed twice with PBS and afterward fixed with 4% formaldehyde in PBS. After washing with PBS, the cells were stained (40 mM citric acid/phosphate buffer pH 6.0, 150 mM NaCl, 2 mM MgCl_2_, 5 mM potassium ferrocyanide, 5 mM potassium ferricyanide, and 0.1% X-Gal) overnight at 37°C. The cells were then washed with PBS and overlaid with 70% glycerin. Micrographs were acquired and analyzed using ImageJ software.

### Cell Transfection

The pinX1 siRNA transient transfection (GGA​GCT​ACC​ATC​AAT​AAT​G) was designed to decrease pinX1 expression temporarily according to a previous publication (Tian et al., 2017). All transfections were performed by Lipofectamine 2000™ (Invitrogen) according to the manufacturer’s instructions.

### Animals

A total of 30 male-specified pathogen-free C57BL/ 6N mice (6–8 weeks) were purchased from Hunan Slack Jingda Experimental Animal Co., Ltd [Certificate of Conformity: no. SCXK (Xiang) 2019–0004; Chongqing, China]. The experimental protocol was approved by the Dalian Medical University Animal Care and Ethics Committee (approval no. 2019–4015), and the animal care and treatment were conducted in accordance with the National Institutes of Health Guide for Care and Use of Laboratory Animals (Publication No. 85-23, revised 1985). The mice were housed one per cage, with all the animals housed in a room at 25°C with 60% humidity and 12 h light/dark cycles. The mice were fed a pelleted diet *ad libitum* with free access to water.

### Establishment of the Animal Model and Administration

Mice were injected subcutaneously over the scapula with 200 μl of suspended tumor cells (2.5×10^5^ cells/mouse) as described previously. The mice were randomly divided into two groups: the tumor-bearing group (control) and tumor-bearing combined with hesperidin (100 mg/kg per day) group (hesperidin). On the third day, after confirming the success of the tumor-bearing, all mice were randomly divided into two groups. One group was given hesperidin by gavage, and the other group was given the same volume of saline. Tumor size, body weight, food intake, and general state of the mice were monitored once a week, and the mice were sacrificed on the 25th day. Samples were taken on the 25th day after tumor-bearing, and the volume and weight of the tumor were recorded.

### Hematoxylin and Eosin (HE) Staining

The liver and kidney were fixed with 4% paraformaldehyde solution overnight, dehydrated in xylene followed by steps of ethanol from 60 to 100%, and then embedded with paraffin. Serial 4-µm sections were cut. The sections were stained with hematoxylin and eosin at room temperature for histologic analysis using a light microscope.

### Western Blot

Cells of each group were collected in a lysis buffer containing 1% PMSF and then quantified by a bicinchoninic acid protein assay kit (Beyotime Institute of Biotechnology, Jiangsu, China). Protein (20 μg) was separated by 8–20% SDS-PAGE and then transferred onto PVDF (Millipore, Bedford, MA, United States) membranes. The PVDF membrane was probed which was quantified by using the multispectral imaging system (Bio-Rad, Hercules, CA, United States).

### Statistical Analysis

Data are expressed as the mean ± SD. Comparisons between two groups were performed using unpaired Student’s t-test. To compare more than three groups, one-way ANOVA followed by Tukey’s *post hoc* test was performed. The data were analyzed using SPSS (version 19.0; IBM Corp). *p* < 0.05 was considered a statistically significant difference.

## Results

### PinX1 Expression and Clinicopathological Features in Patients With SCC

**FIGURE 1:**
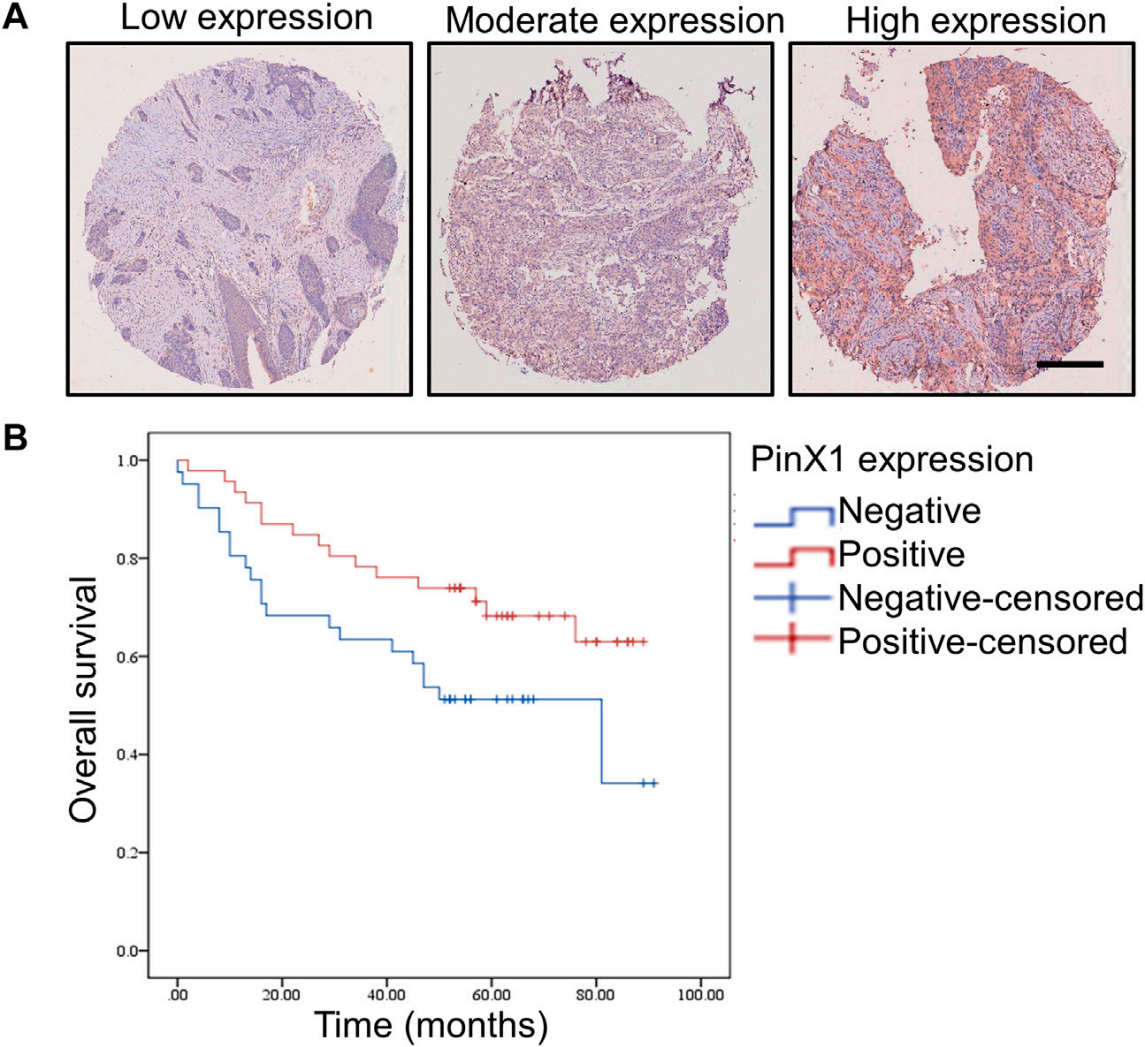
PinX1 expression and the influence on overall survival in human SCC. **(A)** PinX1 expression in human SCC. Scale bar = 200 μM. **(B)** Negative pinX1 expression was correlated with poor overall survival.

An SCC tissue microarray with 86 cancer samples was stained by the pinX1 antibody. The representation pictures are shown in Figure 1A. The PinX1 protein was highly expressed in 46 of the 86 (53.49%) lung cancer tissues, while the low expression of pinX1 was observed in 77 of the 86 (89.53%) adjacent samples (Table 1). The pinX1 expression was correlated with sex (*p* = 0.015, increased in men), grade (*p* = 0.000, increased in grade I/II), T stage (*p* = 0.020, increased in T1/T2), and TNM stage (*p* = 0.025, increased in Ι/II). Moreover, the pinX1 expression was not associated with age, N stage, and M stage (Table 2).

**TABLE 1:** Differential expression of pinX1 in lung cancer and adjacent tissues.

	n	pinX1 expression		Chi-square value	*p*-value
		Positive	Negative		
Lung cancer	86	46	40	27.425	0.000
Adjacent tissues	86	9	77		

a Statistically significant (*p* < 0.05).

**TABLE 2:** Correlation between pinX1 expression and clinicopathological characteristics.

	Variable	pinX1 expression		Total	χ^2^	*p*-value
		Low	High			
Age (year)					0.794	0.095
	≤60	19	10	29		
	>60	27	30	57		
Sex					5.952	0.015
	Female	0	5	5		
	Male	46	36	82		
Grade					14.004	0.000
	I/II	45	28	73		
	III	1	13	14		
T stage					5.372	0.020
	T1/T2	37	26	63		
	T3	5	13	18		
N stage					0.323	0.570
	N0	27	21	48		
	N1	18	18	36		
M stage					1.135	0.287
	M0	46	40	86		
	M1	0	1	1		
TNM stage					5.019	0.025
	Ι/II	37	26	63		
	III/IV	6	14	20		

### The Relationship Between pinX1 Protein Expression and Survival in Patients With SCC

Kaplan–Meier survival curves were used to compare the overall survival rates with negative (<5%) or positive (>5%) pinX1 expression level. The survival analysis showed that a negative pinX1 expression is correlated with poor overall survival (*p* = 0.034, Figure 1B). PinX1 expression, grade, age, N stage, and TNM stage were related to survival (Table 3) according to the univariate Cox regression analysis.

**TABLE 3:** Univariate and multivariate analyses of the factors correlated with overall survival of lung carcinoma patients.

Variable	Univariate analysis			Multivariate analysis		
	Hr	95% CI	*p*-value	Hr	95% CI	*p*-value
Expression	2.023	1.037–3.949	0.039	1.283	0.576–2.858	0.541
Sex	0.847	0.259–2.768	0.784			
Grade	3.234	1.641–6.376	0.001	1.898	0.863–4.174	0.111
Age	1.053	1.011–1.097	0.012	1.051	1.004–1.101	0.034
T stage	1.539	0.974–2.431	0.065			
N stage	2.264	1.422–3.605	0.001	1.917	0.995–3.694	0.066
M stage	5.867	0.771–44.621	0.087			
TNM stage	1.658	1.080–2.545	0.021	2.075	0.954–4.510	0.978

a Statistically significant (*p* < 0.05).

### PinX1 May Be a Key Target to Extend the Patient’s Survival Time in Lung Cancer

**FIGURE 2:**
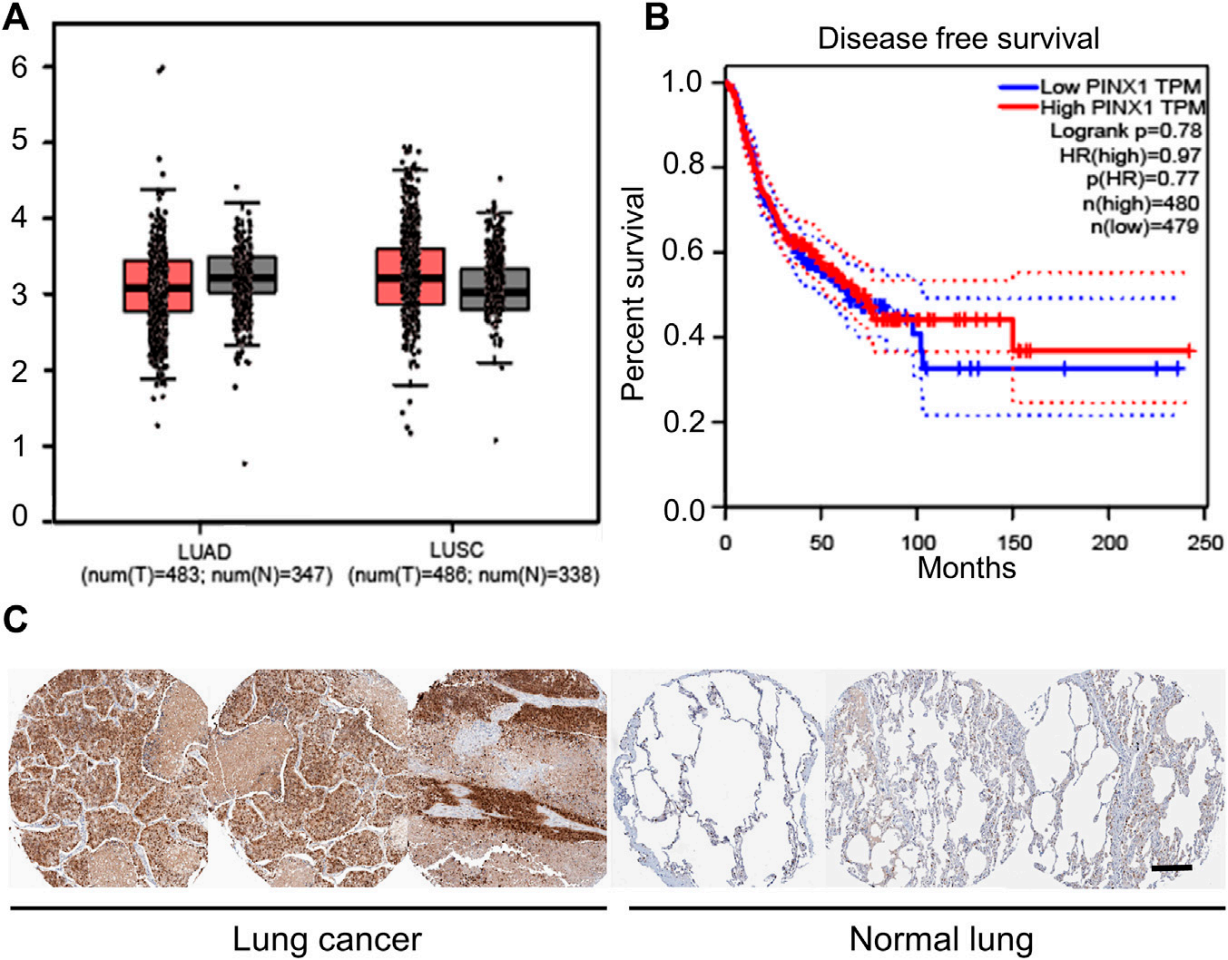
PinX1 may be a key target for chemoprevention of lung cancer. **(A)** PinX1 expression in LUSC and LUAD by the Gene Expression Profiling Interactive Analysis (GEPIA). **(B)** Relation between pinX1 expression and survival time. **(C)** Representative pinX1 expression in the lung and lung cancer from the Human Protein Atlas (HPA) database. Scale bar = 200 μM.

Only SCC patients were collected in the tissue chip. However, non-small-cell lung cancer (NSCLC) can be mainly divided into lung adenocarcinoma (LUAD) and lung squamous cell carcinoma (LUSC). In order to explore the role of pinX1 in exact lung cancer, the role of pinX1 was investigated using a shared database. First, the pinX1 expression level was measured in lung cancer as shown in Figure 2A by the Gene Expression Profiling Interactive Analysis (GEPIA) (http://gepia.cancer-pku.cn/; accessed on 16 July 2021). The pinX1 expression level in LUSC is slightly lower than that in normal lung tissues, but in LUAD, it is slightly higher than that in normal lung tissues. Moreover, the high pinX1 is correlated to longer survival time (Figure 2B). Furthermore, the Human Protein Atlas (HPA) database (https://www.proteinatlas.org/; accessed on 16 July 2021) was used to determine the expression level of pinX1. In HPA, we found that the expression of pinX1 was poor in normal lung tissues, whereas the staining intensity was moderate or strong in lung cancer (Figure 2C). The aforementioned results indicated that pinX1 may be a key target for the chemoprevention of lung cancer to extend the patient’s survival time, which is according to the data we got from the patients’ SCC tissue chip.

### PinX1 Inhibited Cell Proliferation, Translocation, and Invasion and Promoted Cell Senescence

**FIGURE 3:**
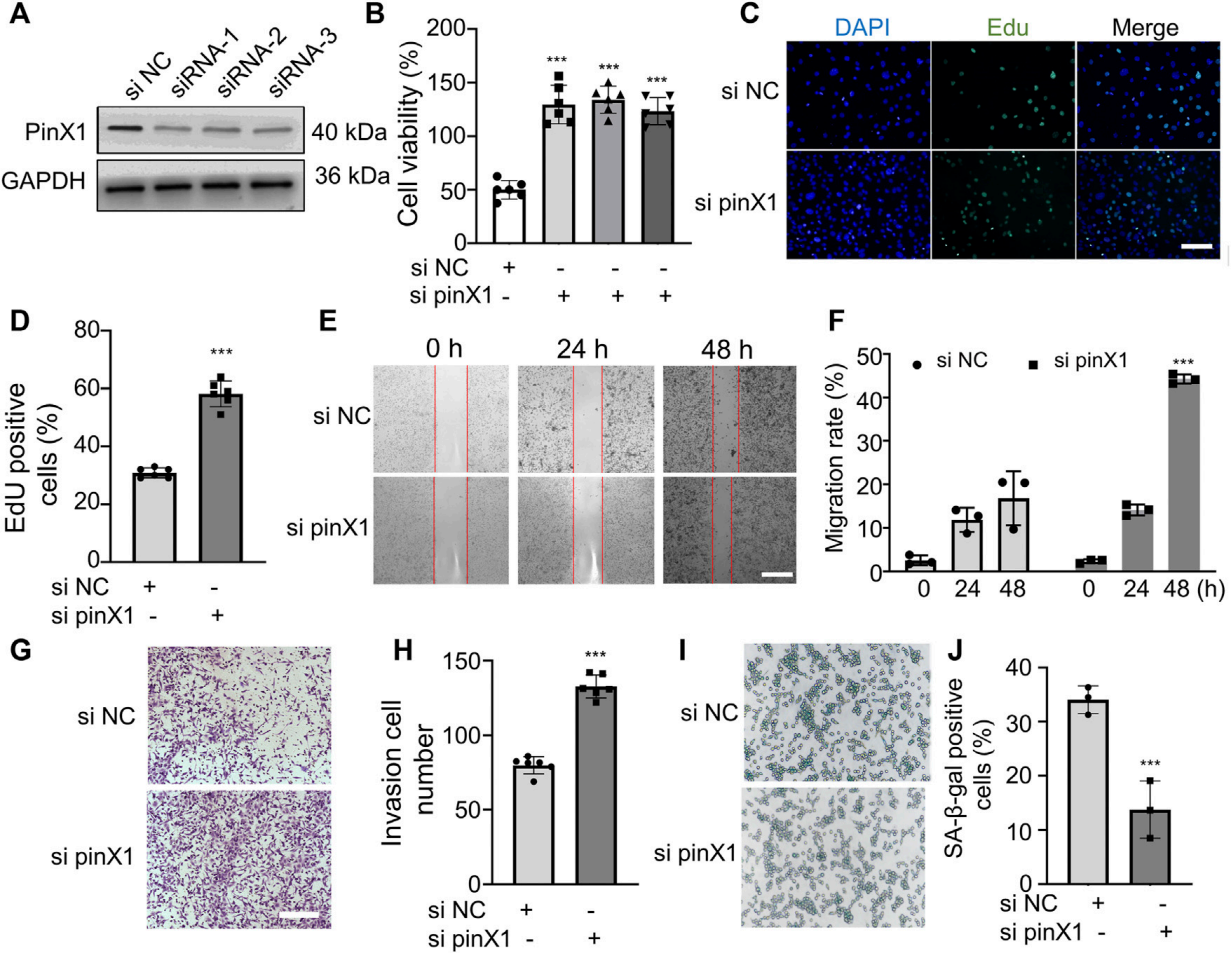
Lack of pinX1 protein expression promoted LLC cell proliferation, invasion, and migration and inhibited cell senescence. **(A)** PinX1 expression. **(B)** Cell viability assay. **(C)** and **(D)** EdU staining. **(E)** and **(F)** Wound healing assay. **(G)** and **(H)** Transwell invasion assay. **(I)** and **(J)** SA-β-gal staining. *n* = 3 or 5; data are shown as mean ± SD. ***p < 0.001 compared with si NC.

In order to investigate the role of pinX1 in lung cancer, the pinX1 expression was knocked down by its specific siRNA *in vitro*. PinX1 was successfully knocked down (Figure 3A), and the knockdown of pinX1 significantly increased cell viability measured by CCK-8 assay (Figure 3B). Moreover, the knockdown of pinX1 significantly promoted LLC cell proliferation (Figure 3C), migration (Figure 3D), and invasion (Figure 3F). Moreover, pinX1 depletion inhibits LLC cell senescence (Figure 3F). The aforementioned result indicated that pinX1 is a key molecule in regulating the malignant behavior of cancer cells.

### Hesperidin Inhibited pinX1 Protein Expression in a Concentration-Dependent Manner

**FIGURE 4:**
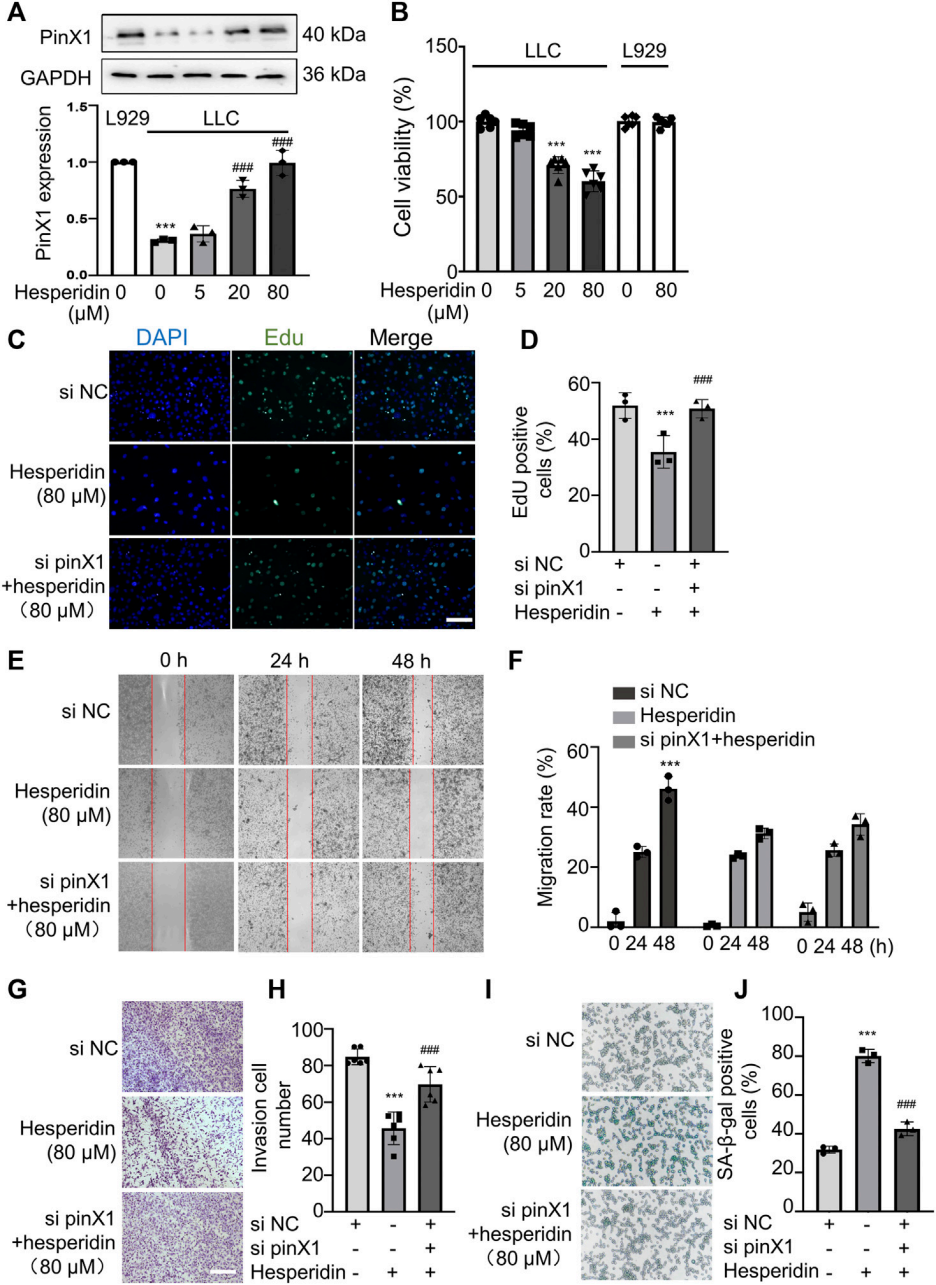
Hesperidin inhibited LLC cell proliferation, invasion, and migration and promoted cell senescence *via* pinX1. **(A)** Hesperidin increased the pinX1 protein expression. *n* = 3; data are shown as mean ± SD. ***p < 0.001 compared with L929 and ###*p* < 0.001 compared with LLC control. **(B)** Hesperidin inhibited cell viability in LLC cells but not L929 cells. n = 5; data are shown as mean ± SD. ***p < 0.001 compared with the control group. **(C)** and **(D)** EdU staining. *n* = 3; data are shown as mean ± SD. ***p < 0.001 compared with the si NC group and ###*p* < 0.001 compared with the hesperidin group. **(E)** and **(F)** Wound healing assay. *n* = 3; data are shown as mean ± SD. ***p < 0.001 compared with the si NC group. **(G)** and **(H)** Transwell invasion assay. *n* = 6; data are shown as mean ± SD. ***p < 0.001 compared with the si NC group and ###*p* < 0.001 compared with the hesperidin group. **(I)** and **(J)** SA-β-gal staining. *n* = 3; data are shown as mean ± SD. ***p < 0.001 compared with the si NC group and ###*p* < 0.001 compared with the hesperidin group.

In order to investigate whether hesperidin regulates pinX1 protein expression, pinX1 expression after hesperidin administration was detected by Western blot. The pinX1 protein expression in normal lung epithelioid fibroblasts, L929 cell line, is higher than that in LLC cells, and hesperidin significantly increased the pinX1 protein expression in a concentration-dependent manner (Figure 4A). Hesperidin inhibits the cell viability of LLC cells in a concentration-dependent manner (in the first four columns) but does not affect the cell viability of L929 cells (Figure 4B), a normal epithelioid fibroblast cell line, which is employed to show that it has no cytotoxic effect on normal cells as indicated in the previous study (Rajamma et al., 2020; Jamali et al., 2021). The aforementioned results indicated that hesperidin is a potential anti-tumor candidate with less lethality on normal lung cells.

### Hesperidin-Induced Inhibition on LLC Cell Proliferation, Translocation, Invasion, and Cell Senescence Was Blocked by pinX1 Knockdown

Based on the aforementioned results, we think hesperidin (80 μM) decreasing cell viability is mostly dependent on inhibiting tumor cell proliferation but does not exert cytotoxic effects. From the perspective of clinical use or future application, choosing the concentration at 80 μM for further investigation on migration and invasion assay is reasonable, and many studies have performed the same (Chen et al., 2022; Jiang et al., 2022; Li et al., 2022) Moreover, in the cell viability assay, cells were in a logarithmic growth phase, and in the wound healing assay, cells were in a plateau phase. Cells in this phase did not proliferate, and we added 2% FBS to maintain the basic metabolism of the cells. Based on this condition, it is more suitable to evaluate the drug’s effect on cell motility which can attenuate the effect of cells on proliferation. Hence, hesperidin (80 μM) was chosen for further investigation on cancer cell invasion and migration. Hesperidin significantly inhibited LLC cell proliferation, and the inhibitory effect of hesperidin can be blocked by pinX1 siRNA (Figure 4C). Similarly, the inhibitory effect of hesperidin on cancer cell invasion (Figure 4D) and migration (Figure 4E) can be canceled by pinX1 siRNA. Also, pinX1 knockdown significantly inhibited cell senescence which is promoted by hesperidin (Figure 4F). These results indicated that the anti-tumor effect of hesperidin is at least partly exerted by upregulating pinX1.

### Hesperidin Inhibited Lung Cancer *In Vivo*

**FIGURE 5:**
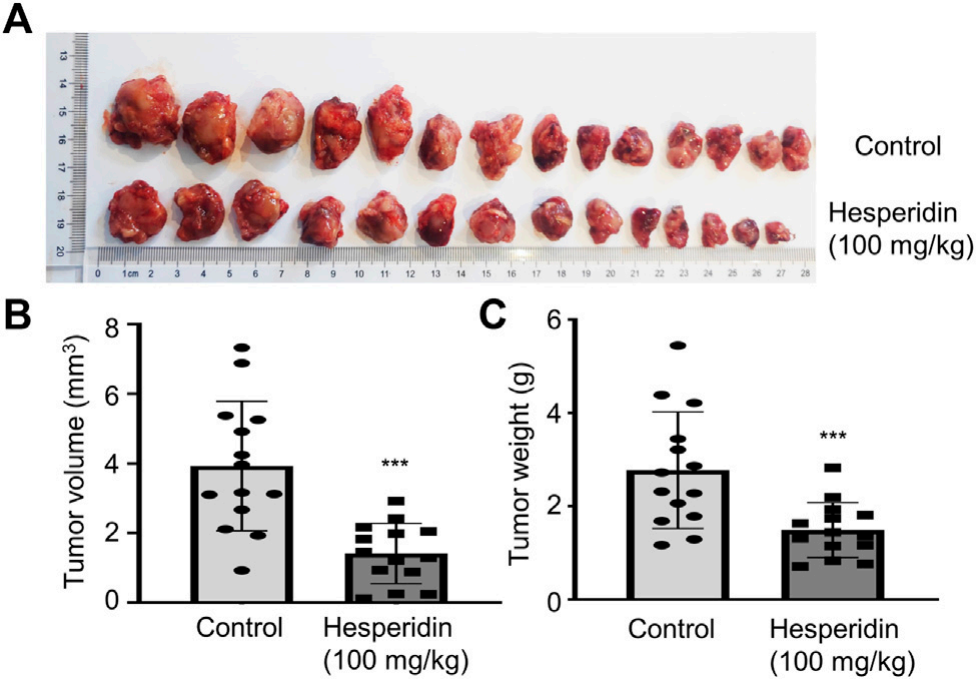
Hesperidin inhibits tumor growth in tumor-bearing mice. **(A)** General photos. **(B)** Tumor volume. **(C)** Tumor weight. n = 14, ***p < 0.001 compared with control mice.

Based on the *in vitro* anti-tumor effect of hesperidin, we think hesperidin may be a potent compound for treating lung cancer. Thus, we evaluated the treatment effect of hesperidin in an *in vivo* tumor-bearing model. In mice that subcutaneously received LLC cells, the tumor burden rapidly increased from the 8th day in the absence of drug intervention, while tumor growth was significantly inhibited after hesperidin administration (Figure 5A). During the experiment, none of the mortality and macroscopic metastases were found upon gross visual examination of the livers, lungs, hearts, and kidneys of the tumor-bearing mice treated with or without hesperidin. When the experiment was complete, tumor weight and tumor volume were recorded as shown in Figure 5, where it was shown that hesperidin significantly reduced tumor volume (Figure 5B) and tumor weight (Figure 5C). The results suggested that hesperidin can effectively inhibit tumor growth *in vivo*.

### Hesperidin Shows No Toxicity While Anti-Tumor

**FIGURE 6:**
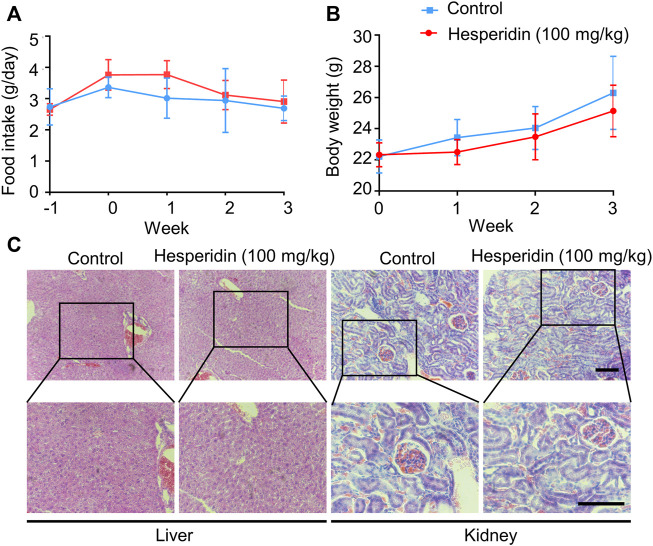
Safety of hesperidin in the treatment of lung cancer. **(A)**. Food intake. **(B)** Body weight. **(C)** Representative of liver and kidney H and E staining. Scale bar = 200 μM.

In the *in vivo* study, we chose a relatively high dose (100 mg/kg) of hesperidin in this investigation based on a previous study (Muhammad et al., 2019). The general state of mice, body weight, and food intake were recorded. As shown in Figures 6A,B, compared to the tumor-bearing group, body weight and food intake did not differ from the mice in the hesperidin-treated group. Furthermore, after the experiment was completed, the mice were dissected, and there was no visible pathological damage in all tissues and organs. Livers and kidneys from the mice treated with or without hesperidin were employed to measure the potential toxicity of hesperidin by H and E staining, which also showed no abnormal changes (Figure 6C). The aforementioned results also indicated that hesperidin is safe at the dose of 100 mg/kg.

## Discussion

Lung cancer is a significant health problem worldwide and current therapeutic strategies lack a sufficient level of specificity and may harm adjacent healthy cells. Finding new drugs or active leads with high efficiency and low toxicity is needed. Hesperidin is a natural product that exhibits anti-tumor effects in various types of cancer and is used in traditional Chinese medicine. In this study, we found that hesperidin inhibits lung cancer *in vitro* and *in vivo* via pinX1.

Hesperidin is a kind of flavonoid from citrus species with antioxidant potential (Ahmed et al., 2022). Its anti-tumor effect has been evaluated in multiple cancer species. Its chemotherapeutic and chemosensitising activities in breast cancer have been reported (Yap et al., 2021). Moreover, the co-administration of hesperidin with doxorubicin, letrozole, or tamoxifen can enhance the efficacies of these clinically available agents (Yap et al., 2021). However, how hesperidin acts on lung cancer and the underlying mechanism need to be clear.

We first evaluated the effect of hesperidin on lung cancer *in vitro*. LLC, a cell line established from the lung of a C57BL mouse bearing primary Lewis lung carcinoma, and a normal mouse fibroblast L929 were employed in our study. We found that hesperidin significantly inhibited LLC cell proliferation, migration, and invasion, which is similar to the previous data reported on breast cancer (Kongtawelert et al., 2020). However, we first reported that hesperidin can induce cancer cell senescence via senescence-associated *β*-galactosidase staining. The main features of senescence cells include stable cell cycle arrest and lack of proliferation-related markers such as Ki67, BrdU, or EdU, since these markers are not only limited to senescent cells but also present in undifferentiated somatic cells, and additional markers should be used to confirm the senescent phenotype. In our study, we observed the lack of proliferation-related markers EdU and increased senescence-associated *β*-galactosidase activity. Hesperidin markedly increased the number of senescence cells. There are some reports of the cell cycle arrest-inducing ability of hesperidin in luminal and triple-negative BC cell lines. In a study, Kabała-Dzik et al. (2018) reported that hesperidin (50–100 μM) for 24–48 h can induce a significantly increased cell population in the G0/G1 phase and G2/M phase in MDA-MB-231 cells and MCF-7 cells. At a relatively high concentration, hesperidin 100 μM induced a high accumulation of MCF-7 cells in the G0/G1 phase, reflecting a G0/G1 phase arrest (Magura et al., 2020). These effects provide clues and ideas for hesperidin to promote cell senescence.

PinX1 is a TRF-binding protein, and it was reported in various cancers. As indicated in a previous study, pinX1 regulated cell progression via PI3K/AKT, MAPK, and *β*-catenin pathways (Kang et al., 2021). Normally, telomeres shorten as the number of mitotic divisions increases, causing cellular senescence. Telomerase can use itself as a template to synthesize telomeres and inhibit cell senescence, and pinX1 is a strong telomerase inhibitor. Telomerase reverse transcriptase (TERT) promoter mutation has been found in human hepatobiliary, pancreatic, and gastrointestinal cancer cell lines (Hirata et al., 2022). The high frequency of hotspot mutations (C228T or C250T) in the promoter region of telomerase reverse transcriptase TERT that encodes the TERT protein has also been found in myxoid liposarcoma (Kunieda et al., 2021). PinX1, known as a potent TERT inhibitor, also contributes to cellular aging and cancer tumorigenicity. Interestingly, the pinX1 protein expression in lung cancer patients is positively related to overall survival time, which is in accordance with a study on thyroid cancer. The knockdown pinX1 expression by specific siRNA significantly reduced cell proliferation rates, colony formation capacity, and cell cycle progression (Kang et al., 2021). Thus, the upregulated pinX1 protein expression may contribute to inhibiting tumor growth. Then, we measured the role of hesperidin on the pinX1 protein expression. As predicted, hesperidin significantly increased the pinX1 protein expression, and the knockdown of pinX1 protein expression by its specific siRNA can at least partially block the inhibitory effect of hesperidin on LLC cell proliferation, migration, and invasion. Also, the effect of hesperidin on inducing cell senescence is attenuated by pinX1 siRNA. The results indicated that pinX1 is an important target of hesperidin.

The *in vivo* efficacy of phytochemicals is highly dependent on their bioavailability. Hesperidin has demonstrated limited bioavailability for its poor water solubility which can limit absorption greatly (Jiao et al., 2020). The anti-tumor effect of hesperidin *in vivo* is poorly reported. A study demonstrated that hesperidin inhibits tumor volume in a xenograft mice model of human osteosarcoma cells (Du et al., 2018). In our study, a relatively high dose of hesperidin is used, and hesperidin significantly decreased tumor volume. It is important to emphasize that we have eliminated the individual differences by increasing the number of tested animals. The results indicate that hesperidin holds a weak anti-tumor effect *in vivo*. Therefore, we believe that the combined application of hesperidin and chemotherapeutic drugs is more promising.

In addition, long time uses, safety, and bioavailability of hesperidin are a rigorous strategy to be solved. Although several toxicity studies have suggested the low risk and safety of hesperidin, little is known about the safety profile of hesperidin. Thus, in this study, we evaluated safety during the experimental period. Promisingly, hesperidin shows no toxicity on the liver and kidney. However, extensive preclinical toxicity studies are required before these compounds can be considered for further drug development and clinical applications.

In brief, our study shows that hesperidin can inhibit LLC cell proliferation, migration, and invasion and induce senescence via pinX1. Some limitations of our study should be mentioned. Based on a previous study, the expression level is relatively low in human H520 and A549 cell lines (Wang et al., 2017). It is also indeed important to detect the pinX1 expression profile in other lung cancer cell lines and to verify the broad applicability of the anti-lung cancer effect of hesperidin. We found that hesperidin can regulate pinX1 expression, but the direct target of hesperidin remains unclear. Moreover, we use pinX1 siRNA to investigate the underlying mechanisms, and a similar experiment *in vivo* based on pinX1 transgenic mice will provide more clues for the study of the mechanism of hesperidin against lung cancer.

## Data Availability

The raw data supporting the conclusion of this article will be made available by the authors, without undue reservation.
